# A New Method for E-Government Procurement Using Collaborative Filtering and Bayesian Approach

**DOI:** 10.1155/2013/129123

**Published:** 2013-12-09

**Authors:** Shuai Zhang, Chengyu Xi, Yan Wang, Wenyu Zhang, Yanhong Chen

**Affiliations:** School of Information, Zhejiang University of Finance and Economics, Hangzhou 310000, China

## Abstract

Nowadays, as the Internet services increase faster than ever before, government systems are reinvented as E-government services. Therefore, government procurement sectors have to face challenges brought by the explosion of service information. This paper presents a novel method for E-government procurement (eGP) to search for the optimal procurement scheme (OPS). Item-based collaborative filtering and Bayesian approach are used to evaluate and select the candidate services to get the top-*M* recommendations such that the involved computation load can be alleviated. A trapezoidal fuzzy number similarity algorithm is applied to support the item-based collaborative filtering and Bayesian approach, since some of the services' attributes can be hardly expressed as certain and static values but only be easily represented as fuzzy values. A prototype system is built and validated with an illustrative example from eGP to confirm the feasibility of our approach.

## 1. Introduction

In this rapidly changing information era, traditional government systems will not be able to meet the requirements of a new age sufficiently. The fast development of Internet, information, and communication technologies make's E-government possible to deliver efficient and cost-effective services, information, and knowledge [1].

E-government procurement (eGP) is an essential one of E-government programs and it is governments' responsibility to have the procurement schemes be supervised by the citizens or the taxpayers; that is, eGP enables active transparency and favors efficient vendor relationships [2]. Simultaneously, eGP systems increase productivity of day-to-day procurement activities within agencies and may attract new suppliers to do business with the government [2]. Unlike E-commerce or enterprise procurement, (1) eGP needs normalization, routinization, and rigorous attitudes more than personal E-commerce; (2) eGP aims at bringing transparency and ultimately reducing corruption [3]; (3) dissimilar to E-commerce or enterprise procurement, the cost of government procurement could be certain, and procurement sectors need not reduce the cost as much as possible.

Therefore, applicable methods should be proposed to meet above issues brought by eGP systems so as to achieve the optimal procurement scheme (OPS). However, the state-of-the-art literature has paid little attention to excogitating an available algorithm to achieve cost-saving, service level optimized, efficient, and effective procurement scheme. In this paper, we propose a novel approach based on collaborative filtering and an extended Bayesian approach to assist the procurement sector in obtaining the OPS. We use item-based collaborative filtering to get the top-*M* recommendations since collaborative filtering is extensively studied and applied to recommender systems and enable user-generated opinions to be exploited in a sophisticated and powerful way [4]. A trapezoidal fuzzy number similarity algorithm is adopted to calculate the similarity between two services so as to extend the item-based collaborative filtering and our initial Bayesian approach [5].

The rest of this paper is arranged as follows. We discuss related works in Section 2. Outline of the proposed algorithm in this paper will be summarized in Section 3 which contains three phases: data preparation stage, filtering stage, and search stage. In Section 4, a prototype system will be built and validated with an illustrative example to verify the performance of our approach.  Section 5 will be the conclusion and some related further works are presented.

## 2. Related Works

### 2.1. E-Government Procurement

E-Government procurement, defined as a procurement method that government sectors utilize to purchase services from vendors via modern information and communication technologies, especially, Internet technologies, is the most important experimental project in the electronic business [6]. Since eGP enables active transparency and corruption reduction and favors efficient and effective vendor relationships, it is gradually becoming an essential part of government programs.

However, along with the increasing new suppliers who are attracted to do business with the government, the explosion of service information makes it difficult for government procurement sectors to set suitable procurement schemes. Therefore, government procurement sectors need to act as active users instead of passive receivers, and the participators of the procedure formulating the procurement scheme should possess some professional knowledge in different industry fields [6] in order to differentiate massive services. Thus, with above preconditions, government procurement sectors are able to search for cost-saving, service level optimized, efficient, and effective procurement schemes that meet their requirements most.

Recent years have seen the incremental interest in eGP by researches focusing on how to establish a proper eGP system to meet eGP's peculiar needs, how to estimate such a system efficaciously, and what index properties should be assessed, and so forth. Concha et al. [2] proposed the e-Government Procurement Observatory Maturity Model to measure the maturity levels of public procurement portals in order to increase the transparency of public procurement and foster better relationships among agencies. Liu and Wang [3] built up a scientific set of evaluation criterion systems on websites of e-government procurement that is suitable for Chinese situation projects. Islam et al. [7] proposed a solution for an eGP system to solve security issues. Jin and Jiang [8] attempted to build up the benefit evaluation system model of eGP and summarized some criteria for the information collection of eGP.

However, up to now, there is still no available algorithm or method that has been proposed to meet the special demands of eGP and help government procurement sectors search for the OPSs. The current paper presents a novel method combining item-based collaborative filtering that has already been extensively applied to provide personalized recommendations and Bayesian approach that has already been widely studied to improve the service level of supply chain management. Considering that searching for the OPS is a highly dynamic process and some of the services' attributes can be hardly expressed as certain and static value, a trapezoidal fuzzy number similarity algorithm is applied to support the item-based collaborative filtering and Bayesian approach.

### 2.2. Item-Based Collaborative Filtering

Collaborative filtering is one of the most successful algorithms which provide recommendations using ratings of users on items [9]. Collaborative filtering consists of user-based and item-based collaborative filtering. Meanwhile, some fusion methods are proposed to improve the accuracy of the personalized recommendations. The item-based collaborative filtering is proposed in view of the reality that active users are more likely to purchase services that are similar to services they have bought before or services they are familiar with.

Therefore, how to properly select similarity weight expression is a significant step of collaborative filtering. Pearson's correlation coefficient and cosine distance [10] are two common expressions proposed as similarity weight. Here, we choose Pearson's correlation coefficient since some previous works conclude that Pearson's correlation coefficient performs better. The formula is as follows:

(1) $$\begin{array}{c} \text{sim}\left(a,b\right)=\frac{\sum_{u\in U_{a}\cap U_{b}}^{}\left(r_{ua}-\bar{r_{a}}\right)\left(r_{ub}-\bar{r_{b}}\right)}{\sqrt{\sum_{u\in U_{a}\cap U_{b}}^{}{\left(r_{ua}-\bar{r_{a}}\right)}^{2}}\sqrt{\sum_{u\in U_{a}\cap U_{b}}^{}{\left(r_{ub}-\bar{r_{b}}\right)}^{2}}}, \end{array}$$

where *a* stands for the target service, *b* represents the services that active users are familiar with or have bought before, *U*_*a*_ is a set of users that have rated target service *a*, *U*_*b*_ is a set of users that have rated service *b*, *r*_*ua*_ represents the rating of the target service *a* that is rated by user *u*, *r*_*ub*_ represents the rating of service *b* that is rated by user *u*, and *r*_*a*_, *r*_*b*_ represent the average rating of service *a* and *b*, respectively.

The second stage is selecting services based on two techniques: the top-*N* technique and threshold selection. According to the top-*N* technique, a predefined number of *N*-best neighbors [11] may be selected as best services. While just meeting this condition is not enough, a selected service should be similar as the expected service; that is, their similarity should exceed a certain threshold value.

After we get the most similar services, we can calculate the weighted average of the rating of the active user *U*_*t*_ on the target service *a* as follows:

(2) $$\begin{array}{c} R_{U_{t}a}=\frac{\sum_{i=1}^{c}\text{sim}\left(a,i\right)\times R_{U_{t}i}}{\sum_{i=1}^{c}\text{sim}\left(a,i\right)}, \end{array}$$

where *c* stands for the number of the most similar services, sim (*a*, *i*) represents the similarity between the target service *a* and the similar service *i*, and *R*_*Uti*_ is the rating of the active user *U*_*t*_ on the similar service *i*.

In this paper, we assume that an active supplier providing *L* services will recommend government procurement sector the top-*M* services using item-based collaborative filtering, so as to narrow the search scope, improve the search precision, and increase the search effectiveness.

### 2.3. Bayesian Approach

In 1763, an English mathematician named Thomas Bayes firstly proposed the well-known Bayesian theorem which is now extensively researched and applied to a large amount of domains, including supply chain management and resource allocation planning towards e-business or e-government.

For example, Narahari et al. [12] proposed a novel Bayesian incentive compatible mechanism for decentralized supply chain formation to elicit the true cost functions, compared with the classical Vickrey-Clarke-Groves mechanisms. This proposed approach reduces supply chain formation's cost significantly on the basis of their experiment. Chen et al. [13] and Lockamy III and McCormack [14] searched for optimal-sourcing strategies under the frameworks of Bayesian models to alleviate supply disruptions that firms have experienced recently. In our previous works, Wu et al. [15] presented a new matrix-based Bayesian approach for recommending the optimal manufacturing resource allocation plan in the area of supply chain management and Zhang et al. [5] sought a time-aware Bayesian approach to find optimal manufacturing service recommendation under the circumstance that manufacturing services are decentralized.

According to our previous Bayesian approach to similarity computation between services, for those services with no historical feedback rating, our previous measure was to set a default value which may not reflect the reality exactly. However, we could not ignore such a phenomenon that if Apple Inc. launches a new product, most of the active users may give high ratings though the new launched product has no historical feedback rating. Therefore, in this study, we extend our initial Bayesian approach by applying the item-based collaborative filtering to predict the ratings of those services without historical feedback rating.

Additionally, in order to boost the effectiveness of searching for the OPS, we also enhance the initial Bayesian approach with fuzzy number similarity algorithm to evaluate the services actively, where the government procurement sector acts as an active user during the eGP process.

### 2.4. Fuzzy Number Similarity Algorithm

The key information required in a multiattribute decision-making (MADM) model includes attribute values, attribute weights, and a mechanism to synthesize this information into an aggregated value or assessment for each alternative [16]. However, in the process of making procurement scheme, the decision makers in government procurement department can not always make completely rational decisions, due to the impossibility of analyzing all the decision information and the limitation of their personal handling abilities. Therefore, decision makers usually need search for the optimal procurement scheme (OPS) instead of the ideal procurement scheme.

In addition, engineering or management decision information is often vague, imprecise, and uncertain by nature [17], as makes it difficult for decision makers to give their assessments on attribute values and weights in crisp values [18]. For this reason, it is reasonable that attributes are defined as fuzzy values; hence, we extend the traditional crisp logic into fuzzy logic [19] to define the attributes in this work.

**Figure 1:**
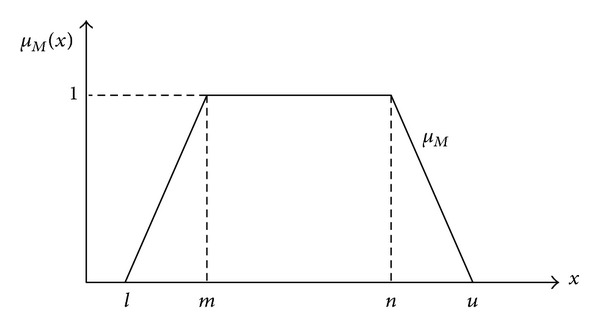
Trapezoidal fuzzy number *M*.

In our proposed method, the criteria will be expressed as trapezoidal fuzzy numbers since trapezoidal fuzzy number offers reasonable description of fuzzy conceptions and easy computations. For instance, *M* = (*l*, *m*, *n*, *u*)  (*u* > *n* > *m* > *l*), shown in Figure 1, is a universally nonnegative trapezoidal fuzzy number. *μ*_*M*_(*x*), which is a real number in the interval [0,1], is a membership function that associates to each element *x* in a universe of discourse *X* [20].

Up to now, many researches [21–24] have proposed numerous algorithms to calculate the similarity between two trapezoidal fuzzy numbers. In this paper, we will not make a detailed list of these algorithms for concision.

## 3. A New Method for E-Government Procurement

Our method is proposed to help the government procurement sector search for the optimal e-procurement scheme which is cost-saving, service level optimized, efficient, and effective. It is found that an OPS usually consists of one or several optimal services under some constraints, so it is helpful to retrieve some candidate services first to expedite the final search process of OPS. Therefore, our approach can be divided into three stages: data preparation stage, filtering stage, and search stage.

In the process of data preparation, criteria for the service evaluation of the services that government wants to procure will be summarized and expressed as trapezoidal fuzzy numbers and the ideal fuzzy values of criteria will be set by decision makers.

In filtering stage, the top-*N* candidate services will be retrieved through threshold elimination, item-based collaborative filtering, and extended Bayesian approach. The threshold elimination method is used to exclude services that do not meet some basic constraints. Item-based collaborative filtering and Bayesian approach are applied to support the two-way selection. During the process of selection, government departments act as active users, while the service providers act as active suppliers.

In the last stage, that is, search stage, if an eGP scheme consisting of *Q* types of services meets several preconditions and constraints, it can be regarded as an OPS.

### 3.1. Data Preparation Stage

This stage consists of two steps: criteria determination and ideal data setting.

#### 3.1.1. Criteria Determination

The evaluation criteria of the services that government needs will be summarized in this stage. In the process of e-procurement, service suppliers do business with government through Web; therefore, it is necessary to combine conventional Quality of Service (QoS) properties with Quality of Web Service (QoWS) parameters while dealing with the service evaluation criteria. Yu and Bouguettaya [25] proposed some typical QoWS parameters: latency, reliability, availability, fee, and reputation. Considering these parameters, we claim that the service evaluation criteria consist of price, performance (ratings provided by historical users), reliability (service life), availability (ability to meet the demands), security, maintainability, maintenance cost, latency time (the reaction time of after-sale service), reputation (ratings on supplier), accuracy (the ability of supplier to send the right services to users) and the level of after-sale service, and so forth.

#### 3.1.2. Ideal Data Setting

Due to the vagueness, imprecision and uncertainty of decision information, and the incomplete rationality of decision makers, it is difficult for decision makers to express their assessments on attribute values and weights in static and specific values. For this reason, it is reasonable that criteria and weights are defined as fuzzy values.

In this step, decision makers of the procurement scheme will set the ideal fuzzy values of criteria to get the ideal procurement scheme, which underlines the OPS using extended Bayesian approach. Because the decision makers possess some professional domain knowledge, the provided ideal fuzzy values will be as rational and reasonable as possible.

### 3.2. Filtering Stage

The two-way selection in this stage is proposed to improve the effectiveness of the service evaluation. The procedure of this selection is as follows. Firstly, the assumed active suppliers recommend government procurement sector the top-*M* services that satisfy the service request. Then the government procurement sector chooses the top-*N* services within *M* services using extended Bayesian approach (*M* ≥ *N*).

#### 3.2.1. Threshold Elimination

For each service request that government needs to purchase, *K* candidate services offered by *J* different suppliers can be found (*K* ≥ *J*). The threshold value expressed as trapezoidal fuzzy number for each criterion set in the data preparation stage should be predefined. Those services whose corresponding attributes' values do not exceed the predefined threshold values, respectively, will be eliminated; therefore, the computation load will be alleviated.

By now, more than 20 ordering methods have been proposed to compare two trapezoidal fuzzy numbers; for example, Li [26] discussed some practical, intelligible, and easily operable approaches. The center of gravity method is one of these approaches, through which the participators need only to compare the values of both the center of gravity and the mean square deviation of the ideal criteria and the corresponding target service's attributes. The following presents the computational formulas of both the center of gravity and the mean square deviation and the comparison rules are shown in Table 1:

(3) $$\begin{array}{c} c\left(M\right)=\frac{\left(u^{2}+n^{2}+un-m^{2}-l^{2}-lm\right)}{\left[3\left(u+n-l-m\right)\right]}, \end{array}$$

(4) σ(M)=[((m4/4)+(l4/12)−(lm3/3))(m−l)+(n3−m3)3  +((n4/4)+(u4/12)−(un3/3))(u−n)  × ((u+n−m−l)2−(c(M))2)−1]1/2,

where *c*(*M*) represents the value of the center of gravity, and *σ*(*M*) represents the value of the mean square deviation. The meaning of *l*, *m*, *n*, and *u* is shown in Figure 1.

#### 3.2.2. Using Item-Based Collaborative Filtering to Get the Top-*M* ****Services

After threshold elimination, there still remain *L* corresponding services offered by suppliers, which satisfy the service request (*K* ≥ *L*). We assume that an active supplier providing all the *L* services will recommend government procurement sector the top-*M* services based on the past ratings or the predicted ratings of government procurement sector on these *L* services.

For those services that have historical ratings from other users but have not been purchased or rated by the active government sector before, by using (1) and (2) for item-based collaborative filtering, the active supplier can predict the possible ratings of government procurement sector on the services.

For those services that have no historical ratings, extended item-based collaborative filtering is proposed to predict their historical ratings. In the initial item-based collaborative filtering, the similarities between the target service and each of the services that active users are familiar with or have bought before are calculated based on the ratings of active users on services. However, in fact, some services may have no historical ratings. Therefore, fuzzy number similarity algorithm is applied to compute the service similarities. Chen [23] proposed a new algorithm for calculating the similarity between two generalized trapezoidal fuzzy numbers *M*_1_ = (*l*_1_, *m*_1_, *n*_1_, *u*_1_) and *M*_2_ = (*l*_2_, *m*_2_, *n*_2_, *u*_2_), and the following formula presents this similarity measure:

(5) sim(M1,M2) =1−|l1−l2|+|m1−m2|+|n1−n2|+|u1−u2|4,

where |*d*| represents the absolute value of the real number *d*.

Since the similarities between corresponding service evaluation criteria of the target service and services that active users are familiar with or have bought before are obtained using (5), then the similarities sim (*S*_1_, *S*_2_) between the target service and the services that active users are familiar with or have bought before can be calculated as follows:

(6) $$\begin{array}{c} \text{sim}\left(S_{1},S_{2}\right)=\sum_{i=1}^{z}w_{i}\times \text{sim}\left(c_{1i},c_{2i}\right), \end{array}$$

where sim (*c*_1*i*_, *c*_2*i*_) stands for the similarities between corresponding service evaluation criteria of the target service and services that active users are familiar with or have bought before, *z* represents the number of service evaluation criteria, and *w*_*i*_ represents the weight of criterion *i* among *z* criteria.

Thus, the active suppliers can also get the ratings of active government sector on the *L* services, even if some of which may have no historical rating. Then, these *L* services can be ranked in the increasing order of their ratings of active government sector, and the top-*M* corresponding services can be recommended to the active government procurement sector (*L* ≥ *M*).

In fact, in this rapidly changing era, most services have no historical rating. On this occasion, the initial item-based collaborative filtering approach may not meet the needs of the situation. Additionally, due to the uncertainty and vagueness of subjective evaluation, the extension for the initial item-based collaborative filtering to fuzzy area is reasonable and practical.

#### 3.2.3. Using Extended Bayesian Approach to Get the Top-*N* ****Services

Our previous work [5] proposed a time-aware Bayesian approach for finding a hypothetical service *S*_*h*_ that satisfies the user's requirement most out of all possible recommended services. It aims to maximize the probability of *S*_*h*_ given the initial service *S*_*i*_. While, in this paper, we try to find the top-*N* optimal services from the previously recommended top-*M* services *S*_*i*_ given an ideal service *S*_*O*_. The following expression shows the conditional probability of each service *S*_*i*_:

(7) $$\begin{array}{c} \arg P\left(S_{i}\;|\;S_{O}\right). \end{array}$$

According to the Bayesian formula, the above expression can be transformed into

(8) $$\begin{array}{c} \arg P\left(S_{i}\;|\;S_{O}\right)=P\left(S_{O}\;|\;S_{i}\right)\times \frac{P\left(S_{i}\right)}{P\left(S_{O}\right)}, \end{array}$$

where *P*(*S*_*i*_) stands for the prior probability of service *i* among the previously recommended top-*M* services, *P*(*S*_*O*_) represents the prior probability of ideal service *S*_*O*_, and *P*(*S*_*O*_ | *S*_*i*_) stands for the conditional probability that *S*_*O*_ would be set as an ideal service when service *S*_*i*_ is an optimal service.

From (8), we can find that if the similarity sim (*S*_*i*_, *S*_*O*_) between each service *S*_*i*_ of the previously recommended top-*M* services and the ideal service *S*_*O*_ is higher, *P*(*S*_*O*_ | *S*_*i*_) tends to be higher. In addition, *P*(*S*_*O*_) is the same for all the top-*M* services *S*_*i*_. Thus, (8) can be transformed into

(9) $$\begin{array}{c} \;\arg P\left(S_{i}\;|\;S_{O}\right)=\underset{}{\arg}\;\text{sim}\left(S_{i},S_{O}\right)\times P\left(S_{i}\right), \end{array}$$

where the similarity sim (*S*_*i*_, *S*_*O*_) between each service *S*_*i*_ of the previously recommended top-*M* services and the ideal service *S*_*O*_ is calculated using (1) or (6).

When analyzing the prior probability *P*(*S*_*h*_) in our previous work, we gave a *default value* to a service with no historical ratings. However, to some extent, this measure may not reflect the reality exactly. Therefore, in this paper, when analyzing the prior probability *P*(*S*_*i*_), we apply the extended item-based collaborative filtering to help predict the historical ratings of the recommended top-*M* services with high probabilities.

According to the increasing order of the conditional probabilities of the previously recommended top-*M* services given the ideal service, government procurement sector can select the top-*N* services.

### 3.3. Search Stage

Through above two stages, government procurement sector can retrieve *N* optimal services for a service request. We define the conditional probability of each of the top-*N* services given the ideal service as the satisfaction degree. However, we may find that not all the services are practically optimal due to their different weights in the OPS and some constraints. Therefore, in the process of searching for the OPS, the above factors need to be taken into account.

For each service request that the government sector needs, there is always an optimal candidate service. However, the procurement scheme consisting of the above optimal candidate services may not be practically optimal. To retrieve anOPS that is cost-saving, service level optimized, efficient, and effective, it must satisfy some preconditions and constraints.

Therefore, we claim that if a procurement scheme *S*_*pi*_ consisting of *Q* optimal services, each of which is among the top-*N* optimal services that satisfy each corresponding service request (total *Q* service requests), satisfies the following constraints, *S*_*pi*_ could be regarded as the OPS:

(10) ∑j=1QC(Sij)≤B,max⁡Pmss(Spi) =max⁡{wP×[∑j=1Qwj×P(Sij ∣ SO)]+wC       ×[1−∑j=1Q(C(Sij)+C(Sij)′)max⁡∑j=1Q(C(Sij)+C(Sij)′)]},0≤Tij−Tij′≤ΔTij,

where *S*_*ij*_ represents the *j*th service of the procurement scheme *S*_*pi*_, *C*(*S*_*ij*_) represents the cost of each service *S*_*ij*_ including postage, while *C*(*S*_*ij*_)′ represents the maintenance charge of each service *S*_*ij*_, *Q* (*Q* ≥ 1) represents the number of services that the government sector needs (specially, if *Q* = 1, the procurement scheme only consists of one service request), *B* represents the budget of the procurement activity, *S*_*pi*_ represents the *i*th possible procurement scheme, *P*_mss_(*S*_*pi*_) stands for the prior probability of procurement scheme *S*_*pi*_ being the optimal scheme, *w*_*P*_ and *w*_*C*_ represent the weight of satisfaction degree and overall cost of services *S*_*ij*_, respectively, when calculating *P*_mss_(*S*_*pi*_), *w*_*j*_ is the weight of service *S*_*ij*_ when calculating *P*_mss_(*S*_*pi*_), *T*_*ij*_ is the delivery time of service *S*_*ij*_ expected by the user, *T*_*ij*_′ is the real delivery time of service *S*_*ij*_, while Δ*T*_*ij*_ is the allowable error time of service *S*_*ij*_ which is predefined, *i* = 1, 2,…,  *N* ^*Q*^, *N* ^*Q*^ is the number of the possible procurement scheme, and *j* = 1, 2,…, *Q*.

Additionally, according to different active users, service *S*_*ij*_ may have to satisfy some particular functional requirements as well.

## 4. An Illustrative Example with the Prototype System

In this section, we will illustrate an example for eGP scheme optimization so as to test the practicality and effectiveness of our proposed approach. The software prototype was developed in C# programming language and ExtJS framework.

**Figure 2:**
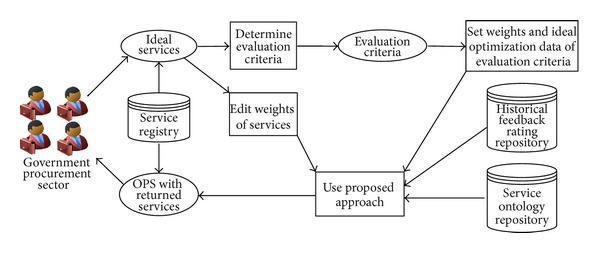
The process of eGP scheme optimization using our proposed approach.

The illustrative example is to find an OPS that has the largest prior probability as the optimal selection in a context with specified ideal services.  Figure 2 shows the process of eGP scheme optimization using our proposed approach. First of all, purchasing staff of the government procurement sector select the ideal services from the service registry. Then choose or input the evaluation criteria of each service, and set weights and ideal optimization data of evaluation criteria. After doing these, the weights of needed services in the procurement scheme must be set. Finally, our proposed approach conducts heuristic searches in the service registry to infer an OPS comprising of one or a few optimal services, which has the biggest prior probability as the optimal selection. The historical feedback ratings and the attributes information of services are extracted from the historical feedback rating repository and the service ontology repository, respectively. Our previous works [27, 28] have already developed a rich body of OWL-based service ontologies which could be a good use for reference to our currently proposed approach.

**Figure 3:**
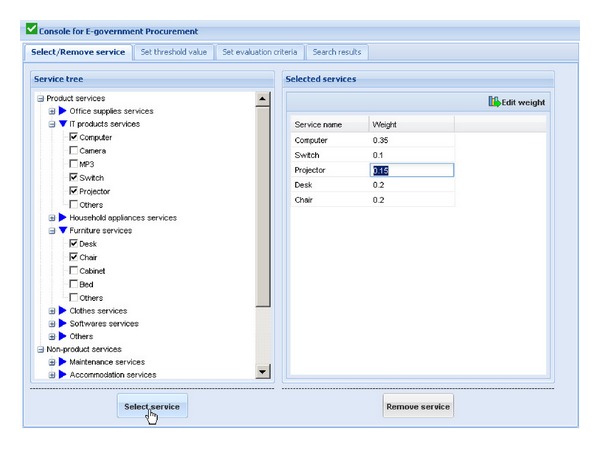
Graphical user interface for procurement scheme optimization system in the prototype system.

Figure 3 shows the graphical user interface for procurement scheme optimization in the prototype system. The left window shows the tree view of services that government may need to purchase and are classified into two categories: product services and nonproduct services. The process of procurement scheme optimization will be shown in the following parts.

**Figure 4:**
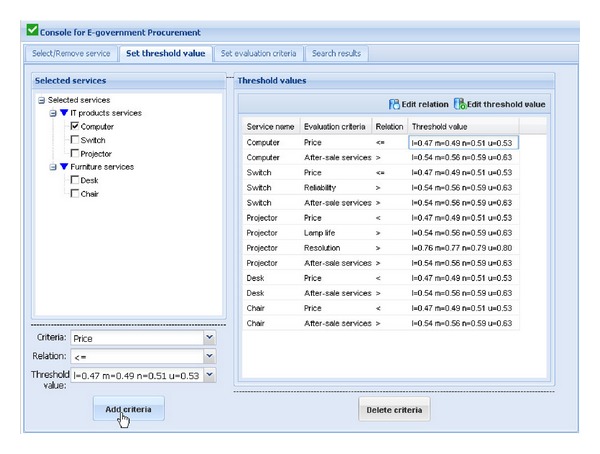
Graphical user interface for setting threshold values of some evaluation criteria of service requests.

**Figure 5:**
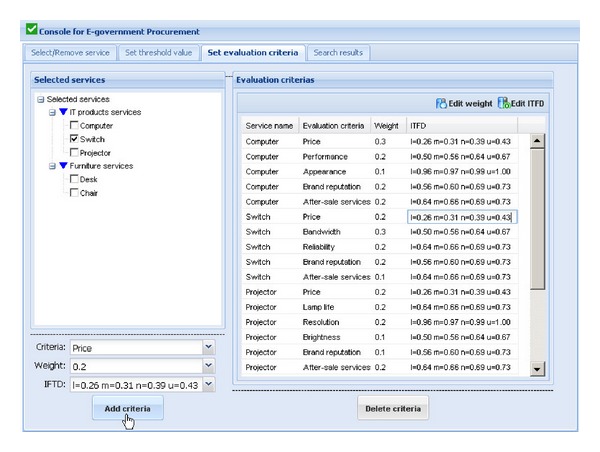
Graphical user interface for setting evaluation criteria of service requests.

**Figure 6:**
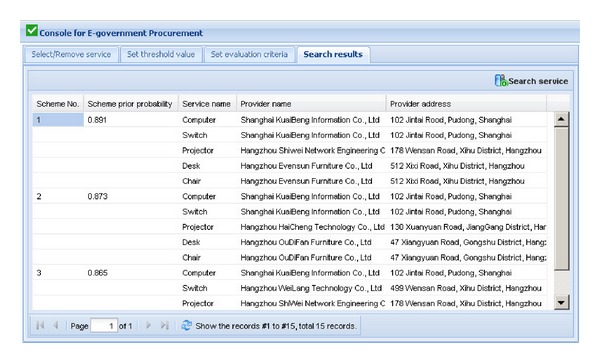
The search results of the illustrative example.

1. We assume that an active user wants to construct an computer room, and s/he wants to purchase computer, switch, projector, desk, and chair. To search for the OPS consisting of above five services, the active user firstly selects the above service requests (required services that the government sector wants to purchase) in the tree view of services in the left window of Figure 3. After finding out all the service requests, the active user clicks on the “Select service” button. The selected services will be shown in the right window of Figure 3, and their weights in this activity of eGP can be edited by the active user. If the wrong services are chosen and shown in the right window, the active user can delete them using “Remove service” button.
2. After selecting the right service requests and setting their weights, threshold values of some evaluation criteria of the service requests should be set by the active user.  Figure 4 shows the graphical user interface for setting threshold values of evaluation criteria of services in this procurement scheme. Choosing each selected service shown in the left window, the active user can set the threshold values expressed as trapezoidal fuzzy numbers of some criteria of the selected service in the drop-down boxes at the bottom of the left window. The setting results will be shown in the right window. The first line of the results “Computer Price ⩽*l* = 0.47  *m* = 0.49  *n* = 0.51  *u* = 0.53” means that the price of the computer that the active user wants must not be higher than average market price which is expressed as trapezoidal fuzzy number since the average market price of the computer is dynamic. For an inexperienced user, due to his/her lack of professional knowledge, s/he can choose threshold values from the corresponding drop-down boxes. While for a power user, s/he can edit new threshold values by clicking on the “Edit threshold value” button in the upper-right corner of the right window. The active user also can reedit the relations using “Edit relation” button.
3. After setting the threshold values of some evaluation criteria of the service requests, the weights in evaluating a service request and the ideal trapezoidal fuzzy data (ITFD) of the evaluation criteria of each service request should be set by the active user.  Figure 5 shows the graphical user interface for setting evaluation criteria of service requests. Choosing each selected service shown in the left window, the active user can set the weights and ITFD of the evaluation criteria of the selected service in the drop-down boxes at the bottom of the left window. The setting results will be shown in the right window. For an inexperienced user, due to his/her lack of professional knowledge, s/he can choose weights and ITFD from the corresponding drop-down boxes. While for a power user, s/he can edit weights and ITFD by clicking on the “Edit weight” button and “Edit ITFD” button, respectively, in the upper-right corner of the right window.
4. Click one the “Search scheme” button in the upper-right corner of the right window of Figure 6, and three top ranked OPSs including scheme No., scheme prior probability (*P*_mss_(*S*_*pi*_)), service name, provider name, and provider address are recommended in an increasing order of prior probabilities, which are shown through the “Search results” tab. The active user usually selects the first OPS with the highest prior probability as 0.891.

## 5. Conclusion

In this paper, we proposed a new method for eGP to search for the OPS. The main contributions of this paper can be summarized as follows:

- development of a methodology for eGP scheme optimization based on item-based collaborative filtering and Bayesian approach, which can deal with the trade-offs among multiple services in an eGP scheme and that among multiple criteria of a service;
- combination of trapezoidal fuzzy algorithm and item-based collaborative filtering and Bayesian approach, which makes it possible to express qualitative descriptions of attributes of services as quantized data and extends the initial item-based collaborative filtering and Bayesian approach to deal with uncertain issues.

However, our proposed approach still has a limitation for large-scale real-world applications. Although there exist many upper-level ontologies and domain-specific ontologies, few service ontologies express the attributes of services as trapezoidal fuzzy numbers. Thus, it is desirable to overcome this limitation in our future works so as to reduce the difficulty of putting our proposed approach into practice.

## Figures and Tables

**Table 1:** Comparison rules for two trapezoidal fuzzy numbers.

The center of gravity	*c*(*M*_1_) > *c*(*M*_2_)	*c*(*M*_1_) < *c*(*M*_2_)	*c*(*M*_1_) = *c*(*M*_2_)		
The mean square deviation	Any type	Any type	σ(*M*_1_) > σ(*M*_2_)	σ(*M*_1_) < σ(*M*_2_)	σ(*M*_1_) = σ(*M*_2_)
Ordering results	*M*_1_ > *M*_2_	*M*_1_ < *M*_2_	*M*_1_ > *M*_2_	*M*_1_ < *M*_2_	*M*_1_ = *M*_2_
